# Correlation between clinical features and magnetic resonance imaging findings in lumbar disc prolapse

**DOI:** 10.4103/0019-5413.77127

**Published:** 2011

**Authors:** Saurabh Jain, Sudhir Kumar

**Affiliations:** *Department of Orthopaedics, University College of Medical Sciences & GTB Hospital, University of Delhi, Dilshad Garden, Delhi, India*

## CITATION

Janardhana AP, Rajagopal, Rao S, Kamath A. Correlation between clinical features and magnetic resonance imaging findings in lumbar disc prolapse. Indian J Orthop 2010;44:3:263-9.

## RESEARCH QUESTION

To study the correlation between the abnormalities observed on magnetic resonance imaging (MRI) and clinical features in patients of lumbar disc prolapse and to know about its significance in decision-making for treatment.

## PATIENT SELECTION AND METHOD

Patients with lumbar disc prolapse were evaluated for pain distribution, neurological symptoms and signs. The clinical criteria used were: a) low backache with radiation to the lower limb, b) radicular pain along a specific dermatome, c) nerve root tension signs like straight leg raising test (SLRT) and d) presence of neurological symptoms and signs. Exclusion criteria included patients with acute pain, relieved with bed rest and analgesics for three weeks and patients who had completely recovered. Dermatomal level of pain distribution and neurological signs and symptoms were recorded and correlated with MR evaluation, which included level of herniation, grades of disc degeneration (Pffirrmann Grade 1 to 5), type of herniation (normal, bulge, protusion, extrusion), neural foramen compromise (thecal sac compression, foramen compromise, nerve root contact, nerve root compression) and miscellaneous findings. Statistical analysis included the Kappa coefficient, Odd’s ratio, and logistic regression analysis.

## FINDINGS

The dermatological pain, neurological symptoms and deficit was seen in 123, 73 and 36 patients respectively. On MRI, disc bulges, protrusion and extrusion was seen in 208, 56 and 26 levels, respectively. Among 66 patients of root compression, 57 were symptomatic with radicular pain while 23 had neurological deficits. Among 31 central disc protrusions and 23 centrolateral, 20 in each group were asymptomatic whereas two with far lateral disc protrusion were symptomatic with deficits. Of 10 having central disc extrusion only five were symptomatic and all 16 centrolateral disc extrusions were symptomatic.

MRI evidence of neural root or foramen compromise with disc bulge, protrusion or extrusion with symptoms had odd’s ratio 41.7 and 6.0 respectively. Patients with gross extrusion and central protrusion and not compromising neural foramen were asymptomatic. There was no statistical correlation between clinical symptoms and MRI findings like disc bulge, disc protrusion and disc extrusion (*P* value 0.013, 0.124 and 0.013 respectively).

## CLINICAL RELEVANCE TO ORTHOPEDIC PRACTICE

Clinical findings correlate well with MRI findings, but all MRI abnormalities need not have a clinical significance. There is significant association between evidence of centrolateral protrusion and extrusion with neural foramen compromise and root compression with clinical symptoms. There is no statistical correlation between clinical symptoms and other MRI findings like central disc bulge, protrusion and extrusion. The multiple level disc lesions and neural foramen compromise is associated with neurological deficits.

## EXPERT OPINION

The study provides valuable information in support of the earlier literature verifying that MRI level of disc prolapse correlates well with the clinical level and MRI may not be essential for clinical diagnosis ascertaining the importance of meticulous clinical examination. MRI is essential when surgery is planned. The study lacks in adding any newer information from previously known facts using a lesser resolution MRI and not mentioning the expertise of clinician for clinical assessment. Further the intra-observer bias in assessment of MRI findings was also not specified.

